# Hepatitis following famotidine: a case report

**DOI:** 10.1186/1757-1626-2-89

**Published:** 2009-01-27

**Authors:** Nishant Gupta, Chirag Patel, Mukta Panda

**Affiliations:** 1 Department of Internal Medicine, University of Tennessee, College of Medicine, Chattanooga, TN 37403, USA

## Abstract

H2 receptor antagonists can rarely cause idiosyncratic drug reactions leading to acute hepatitis. Famotidine, however, is considered a relatively safe drug with regards to hepatotoxicity. We report a case of a 47 year old male with a history of hepatitis C who developed acute hepatitis on the third day of hospitalization with a dramatic rise in his liver enzymes from normal values at the time of admission. The acute rise in liver enzymes made us consider an adverse drug reaction and famotidine was discontinued. Subsequently his liver enzymes came back to normal in seven days. Thus, physicians should consider famotidine induced hepatitis as a possible etiology of acute liver dysfunction.

## Introduction

Drug-induced hepatic injury is a very common cause of hepatitis in adults. Drug hepatotoxicity is the most common cause of fulminant liver failure in the United States [1]. Hepatotoxicity can occur with many drugs through a variety of mechanisms and can present with an array of clinical presentations ranging from asymptomatic mild biochemical abnormalities to an acute illness that resembles viral hepatitis [2,3]. A variety of drugs like acetaminophen, isoniazid, sulfonamides, methotrexate, methyldopa etc. are well known to cause liver damage.

Current H2 receptor antagonists have been very rarely associated with idiosyncratic drug reactions leading to liver failure. In the past, two H2 receptor antagonists have been withdrawn because of high risk of liver toxicity [4,5]. However liver toxicity has never been shown to be a class effect of H2 receptor antagonists [6]. Cimetidine and Ranitidine can produce rare idiosyncratic hepatotoxic reactions [6]. Famotidine, however, is considered to be a relatively safe drug with regards to hepatotoxicity [7]. We report a case of acute hepatic failure secondary to the use of famotidine. To our knowledge, there have only been 4 cases in the English literature where famotidine was responsible for hepatotoxicity [8-10].

## Case presentation

**Figure 1:**
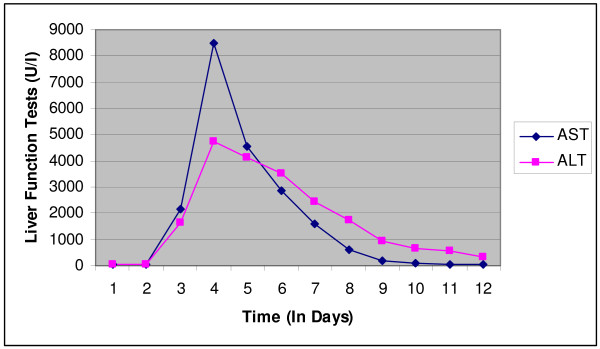
**Graph showing the trend of Liver Function tests in our patient**. Famotidine was stopped on day 4.

A 47 year old male with a past history of asymptomatic chronic Hepatitis C diagnosed 4 years ago, on no home medications, came to the hospital with chief complaints of right upper quadrant abdominal pain and vomiting for one day. His vital signs on admission were stable. On physical examination the abdomen was tender to palpation in right upper quadrant with no palpable organomegaly. He had no stigmata of end stage liver disease. Laboratory data at the time of admission showed WBC 14.5 th/mm^3 ^(normal 4 – 11 th/mm^3^), Hemoglobin 16 g/dl (normal 14 – 18 gm/dl), Platelets 190 th/mm^3 ^(normal 130 – 400 th/mm^3^), Total bilirubin 0.7 mg/dl (normal 0.0 – 1 mg/dl), INR 1.4, total protein 5.3 gm/dl (normal 6.3 – 8.2 gm/dl), Albumin 2.6 gm/dl (normal 3.4 – 5 gm/dl), AST 28 U/l (normal 15 – 37 U/l), ALT 52 U/l (normal 30 – 65 U/l), Alkaline phosphatase 99 U/l (normal 50 – 136 U/l) and a Lipase level of 174 U/l (normal 114 – 286 U/l). An ultrasound of the abdomen done at the time of admission showed a stone in the gall bladder neck with pericholecystic fluid consistent with our clinical diagnosis of acute cholecystitis. The patient was admitted to the hospital, started on Morphine, Cefoxitin and Famotidine and subsequently underwent laparoscopic cholecystectomy the next day. Fentanyl, propofol and vecuronium were used for anesthesia. His hospital course was uneventful until the third day when he started complaining of some abdominal pain with a change in urine color. A complete metabolic panel was obtained on the patient at this time which now showed AST 8466 U/l, ALT 4755 U/l, Total bilirubin 2.5 mg/dl, INR 2.4 with a normal alkaline phosphatase. The sudden elevation in liver enzymes within a couple of days of hospitalization made us think of a possible medication induced adverse reaction. The patient's medications at that time were cefoxitin, morphine and famotidine. Famotidine was discontinued at this time and serial measurements of liver enzymes were done. The liver enzymes started improving from the next day and were back to normal within the next 7 days. (Figure 1) No further investigations were done on the patient as the temporal association of the administration of famotidine and elevation in liver enzymes combined with the response demonstrated after discontinuation of famotidine was very significant in this case.

## Discussion

H2 receptor antagonists are widely used for the treatment of peptic ulcer and gastroesophageal reflux disease. In addition they are commonly prescribed to hospitalized patients for stress ulcer prevention. In general they are very well tolerated drugs and have very few side effects. They can rarely cause adverse hepatic effects which are mostly asymptomatic [11]. While there has been some data regarding possible idiosyncratic reactions to cimetidine and ranitidine leading to acute liver failure, no such association has been reported to date with famotidine. In a case-control study done by Garcia et al [6], rare adverse hepatic reactions were seen with the use of ranitidine, cimetidine and omeprazole but not with famotidine. Studies done by Ohnishi [7] and Luyendyk et al [12] have also shown famotidine to be safe with regards to hepatotoxicity.

To our knowledge there have been four cases reported where famotidine was responsible for hepatic failure [8-10]. In the above mentioned four cases, hepatitis was noted after a few weeks of famotidine. Our case is unique in that the adverse reaction was noted within 36 hours after the administration of famotidine.

There have been various studies which have looked at the pharmacokinetics of famotidine following administration to patients with concomitant liver disease including decompensated cirrhosis [7,13,14]. Most of these studies conclude that famotidine clearance is not altered with impairment of hepatic function. However, one study done by Ohnishi et al [15] showed that famotidine clearance was reduced in decompensated cirrhosis. However, that study also showed that famotidine clearance was affected by renal function. Our patient in addition to being Hepatitis C positive had also developed acute kidney injury at the same time with a serum creatinine rising from 0.8 mg/dl to 2.2 mg/dl (GFR 34 ml/min). His creatinine also returned to baseline of 1 mg/dl at 5 days. The exact significance of this impaired renal function on the development of acute hepatitis is unknown at this time but it could potentially have resulted in a decreased clearance of the drug and thus predisposing him to this adverse reaction. Although just a solitary incidence, it might suggest the need for a larger trial looking at the pharmacokinetics of famotidine in patients with combined renal and hepatic dysfunction.

In conclusion, as famotidine is a commonly used medication both for treatment and prophylaxis, physicians need to be aware of the possibility of this severe idiosyncratic reaction leading to development of acute hepatitis following administration of famotidine especially in patients with hepatic or renal dysfunction and should promptly discontinue the drug if famotidine induced liver toxicity is suspected.

## Consent

Written informed consent was obtained from the patient for publication of this case report and accompanying images. A copy of the written consent is available for review by the Editor-in-Chief of this journal.

## Competing interests

The authors declare that they have no competing interests.

## Authors' contributions

NG analyzed the patient data and wrote the manuscript, CP helped in analysis and interpretation of patient data and MP had a major contribution in writing the final manuscript. All authors have read and approved the final manuscript.
